# HIV self‐testing and oral pre‐exposure prophylaxis are empowering for sex workers and their intimate partners: a qualitative study in Uganda

**DOI:** 10.1002/jia2.25782

**Published:** 2021-09-02

**Authors:** Andrew Mujugira, Agnes Nakyanzi, Vicent Kasiita, Brenda Kamusiime, Grace K. Nalukwago, Alisaati Nalumansi, Chris C. Twesigye, Timothy R. Muwonge, Jared M. Baeten, Monique A. Wyatt, Jessica E. Haberer, Norma C. Ware

**Affiliations:** ^1^ Infectious Diseases Institute, College of Health Sciences Makerere University Kampala Uganda; ^2^ Department of Epidemiology and Biostatistics, School of Public Health Makerere University Kampala Uganda; ^3^ Department of Global Health University of Washington Seattle Washington USA; ^4^ Department of Global Health and Social Medicine Harvard Medical School Boston Massachusetts USA; ^5^ Harvard Global Cambridge Massachusetts USA; ^6^ Massachusetts General Hospital and Harvard Medical School Boston Massachusetts USA

**Keywords:** Africa, empowerment, HIV prevention, HIV self‐testing, pre‐exposure prophylaxis, sex workers

## Abstract

**Introduction:**

HIV self‐testing (HIVST) and oral pre‐exposure prophylaxis (PrEP) are complementary, evidence‐based, self‐controlled HIV prevention tools that may be particularly appealing to sex workers. Understanding how HIVST and PrEP are perceived and used by sex workers and their intimate partners could inform prevention delivery for this population. We conducted qualitative interviews to examine ways in which HIVST and PrEP use influence prevention choices among sex workers in Uganda.

**Methods:**

Within a randomized trial of HIVST and PrEP among 110 HIV‐negative cisgender women, cisgender men and transgender women sex workers (NCT03426670), we conducted 40 qualitative interviews with 30 sex workers and 10 intimate partners (June 2018 to January 2020). Sex worker interviews explored (a) experiences of using HIVST kits; (b) how HIVST was performed with sexual partners; (c) impact of HIVST on PrEP pill taking; and (d) sexual risk behaviours after HIVST. Partner interviews covered (i) introduction of HIVST; (ii) experiences of using HIVST; (iii) HIV status disclosure; and (iv) HIVST's effect on sexual behaviours. Data were analysed using an inductive content analytic approach centering on descriptive category development. Together, these categories detail the meaning of HIVST and PrEP for these qualitative participants.

**Results:**

Using HIVST and PrEP was empowering for this group of sex workers and their partners. Three types of empowerment were observed: (a) economic; (b) relational; and (c) sexual health. (i) Using HIVST and PrEP made sex without condoms safer. Sex workers could charge more for condomless sex, which was empowering economically. (ii) Self‐testing restored trust in partners’ fidelity upon being reunited after a separation. This trust, in combination with condomless sex made possible by PrEP use, restored intimacy, empowering partnered relationships. (iii) HIVST and PrEP enabled sex workers to take control of their HIV prevention efforts and avoid the stigma of public clinic visits. In this way they were empowered to protect their sexual health.

**Conclusions:**

In this sample, sex workers’ use of HIVST and PrEP benefitted not only prevention efforts, but also economic and relational empowerment. Understanding these larger benefits and communicating them to stakeholders could strengthen uptake and use of combination prevention interventions in this marginalized population.

## 1 INTRODUCTION

Worldwide, transgender and female sex workers (SW) are 48.8 and 13.5 times more likely to be living with HIV than other women of reproductive age, respectively [1, 2]. Males who engage in transactional sex are 21 times more likely to be HIV‐positive, compared to the general male population [3]. The World Health Organization (WHO) recommends HIV testing for sex workers, along with linkage to prevention and care services [4]. Yet SW are less likely than the general population to seek HIV services because of concerns about confidentiality, sex work stigma, discrimination from health workers and lack of targeted programmes for SW [5, 6].

HIV self‐testing (HIVST), a process in which an individual performs an HIV rapid diagnostic test and interprets the result in private, is highly acceptable, convenient and discreet and may be empowering for SW not accessing traditional HIV testing services [7, 8]. This emerging strategy has been shown to significantly increase recent and repeat HIV testing among SW [9, 10], and is being scaled up for key populations in high HIV prevalence settings [11]. The WHO also recommends oral pre‐exposure prophylaxis (PrEP) for HIV‐negative persons at substantial and ongoing HIV risk [12]. PrEP effectively prevents HIV acquisition, but requires high adherence; adherence is the main predictor of effectiveness [13]. HIVST and PrEP are complementary self‐controlled prevention tools that could be combined to build self‐efficacy and empowerment and motivate PrEP adherence.

In sub‐Saharan Africa and elsewhere, sex work is often a means to make ends meet and gain financial independence [14]. Financial considerations may take precedence over HIV protection when SW earn more for condomless sex [15]. HIVST and PrEP could be protective for African SW who recognize HIV risk but are unable to use condoms consistently [16, 17]. Studies from Africa suggest HIVST could decrease facility‐based testing barriers such as stigma and discrimination, the logistics of getting to clinic, as well as increase autonomy and self‐empowerment [18, 19]. People with HIV prefer HIVST to standard HIV testing when re‐initiating antiretroviral therapy (ART) [20]. PrEP users, including SWs, may have similar testing preferences when refilling or re‐initiating PrEP. If so, HIVST and PrEP may reinforce each other.

To our knowledge, no published qualitative studies have evaluated how joint HIVST and PrEP use influence prevention choices among SW and their partners. Within a randomized trial designed to assess the impact of HIVST on PrEP adherence, we conducted a qualitative study to examine ways in which HIVST and PrEP use influenced prevention choices among HIV‐negative SW in Uganda.

## 2 METHODS

### 2.1 Population and procedures

The Empower Study was a randomized trial evaluating the effect of HIVST on PrEP adherence and sexual risk behaviours among 110 cisgender women, cisgender men and transgender women SW in Kampala, Uganda (ClinicalTrials.gov: NCT03426670). The study took place between June 2018 and January 2020. HIV‐negative SW were randomized 1:1 to receive monthly HIVST plus quarterly in‐clinic testing (intervention) or quarterly in‐clinic HIV testing alone (standard of care), using block randomization (REDCap, University of Washington) [21, 22], and followed for 12 months. Intervention arm participants were trained in the use of OraQuick HIV self‐test kits (OraSure Technologies, Bethlehem, PA, USA). At each study visit, they received four HIVST kits; two for their own use and two for testing sexual partners (up to 20 kits during the study). PrEP medication was dispensed quarterly, and all participants received three bottles each with a one‐month supply. Because HIVST kit supplies were limited, participants were told to use their own discretion when determining which partners to test, that is, intimate (nonpaying) partners with whom they had ongoing relationships or clients (paying partners). SW were instructed to self‐test monthly during the two months between quarterly visits, before opening each new bottle of PrEP. PrEP adherence was measured using electronic monitors (Wisepill Technologies, South Africa), and dried blood spot samples for quantification of tenofovir diphosphate levels [23]. HIVST use, PrEP adherence and sexual risk behaviours were assessed using semi‐structured questionnaires during monthly phone interviews and quarterly clinic visits. All participants received individualized HIV risk‐reduction counselling, free condoms, and screening and treatment for sexually transmitted infections.

### 2.2 Sampling and recruitment

A random sample of 30 SW in the HIVST arm and 10 intimate partners (persons in ongoing sexual relationships with SW) were invited to participate in the qualitative study. Twelve SW could not be contacted for interviews because of unreachable phone numbers or relocation outside the study catchment area. These 12 were replaced with other trial participants using purposeful sampling; inclusion criteria were use of HIVST, secondary distribution of HIVST to partner and willingness to refer partner for qualitative interviews.

During follow‐up visits for the larger trial, SW qualitative participants provided contact details for 23 partner participants who had used HIVST. Partners were contacted by phone to invite participation; seven declined interviews and six could not be reached (phones unavailable). The remaining 10 were scheduled for in‐depth interviews. All were intimate partners and two had children with the SW participant.

### 2.3 Data collection

Data collection consisted of a single, in‐person qualitative interview with each of 30 participating SW and 10 intimate partners (Total: 40 interviews). Interviews with SW included the following topics: (a) experiences of using HIVST kits; (b) descriptions of how HIVST was performed with sexual partners; (c) the impact of HIVST on PrEP use; and (d) sexual risk behaviours after HIVST. Partner interviews explored: (i) how HIVST was introduced; (ii) experiences of using HIVST; (iii) HIV status disclosure; and (iv) effect of HIVST on sexual behaviours. All interviews were conducted in English or Luganda (local language) by trained research assistants (cisgender women and men) at locations of the participant's choice where conversations could not be overheard (interview guides are included in Appendix S1 and S2). Interviews were about 60‐minute long, audio‐recorded with the participant's permission and transcribed in English by the interviewer from recordings. Quality checks were performed for each transcript, with corrections and revisions made to errors identified. Each participant received an IRB‐approved reimbursement of UGX 30,000 (US $7.86).

### 2.4 Data analysis

We used an inductive, content analytic approach to data analysis [24]. Analysis began with repeated reviews of interview transcripts for content on experiences of HIVST and PrEP use. Open coding, in which relevant content is delineated and provisionally labelled, was carried out to identify specific sections of text. Provisional labels were defined and illustrated to become codes, which were assembled into a codebook. The codebook was used to code the data, with Dedoose software (SCRC, Hermosa Beach, CA, USA) organizing the coding process. At the end of the coding process, codes were used to sort the data to suggest concepts corresponding to HIVST and PrEP use. Content categories were developed from the initial concepts. Each category consisted of a descriptive label, elaborative text and interview quotes illustrating the concept. Categories appear as Qualitative Results, below. They represent all the primary themes identifiable in the data; saturation was achieved. The COREQ checklist was used for reporting study findings [25].

### 2.5 Ethics approval

The study was approved by the Higher Degrees Research Ethics Committee (Makerere University School of Public Health), Partners Human Research Committee (Massachusetts General Hospital) and Uganda National Council for Science and Technology. Each qualitative study participant provided separate written informed consent in English or Luganda.

## 3 RESULTS

### 3.1 Participant characteristics

Twenty‐one SW (70%) were cisgender women (female sex workers; FSW), six self‐identified as transgender women (TGW) and three were men who have sex with men (MSM) (Table 1). Of the 10 intimate partners interviewed, five were partners of TGW, four were partners of FSW and one was an MSM partner. The median age was 25 years for the SW group (interquartile range [IQR] 22 to 29) and 25 years (IQR 23 to 31) for the intimate partner group. Most SW (83%) had ≤11 years of education, 70% had intimate partners and 60% had at least one child. Sex work was the main source of income for 70%, and the median monthly income was UGX 300,000 ($77.70); Uganda average monthly income was UGX 416,000 ($107.74) [26]. Nearly all (97%) reported having access to condoms. The median charge for vaginal and anal sex was UGX 5000 and 50,000 ($1.30 and $12.95) with a condom, and UGX 20,000 and 125,000 ($5.18 and $32.38) without a condom, respectively.

**Table 1:** Baseline characteristics of sex worker participants (*N* = 30)

Characteristic^+^	Frequency (%)
Sex	
Female	21 (70)
Male	3 (10)
Transgender	6 (20)
Age	
18 to 24	12 (40)
25 to 29	10 (33)
30 to 34	5 (17)
>35	3 (10)
Number of completed years of education	
0	2 (7)
1 to 7	10 (33)
8 to 11	13 (43)
12 and above	5 (17)
Marital status	
Divorced/separated/widowed	6 (20)
Married	1 (3)
Single with a regular partner	12 (40)
Single with no regular partner	11 (37)
Number of children	
None	12 (40)
1 to 2 Children	8 (27)
≥3 Children	10 (33)
Average monthly income (Uganda shillings; median (IQR))	300,000 (200,000 to 500,000)
Sex work main source of income	
No	9 (30)
Yes	21 (70)
Charge for vaginal sex with a condom; median (IQR) (*n* = 21)	5000 (5000 to 8000)
Charge for vaginal sex without a condom; median (IQR) (*n* = 13)	20,000 (10,000 to 50,000)
Charge for anal sex with a condom; median (IQR) (*n* = 9)	50,000 (30,000 to 150,000)
Charge for anal sex without a condom; median (IQR) (*n* = 6)	125,000 (50,000 to 350,000)

Abbreviation: IQR, interquartile range. ^a^Quantitative data derived from parent clinical trial.

At enrollment, 97% were willing to use HIVST, 80% worried about getting HIV and 77% believed PrEP could protect them from HIV. All (100%) self‐reported using HIVST at each quarterly visit. Disclosure of sex work to intimate partners was low: 18% and 26% at baseline and month 12, respectively. Overall, 71% reported not disclosing sex work to anyone during the study. Good or excellent PrEP adherence was self‐reported by 65% across all visits.

### 3.2 Qualitative results

Below, we present three content categories representing results of the qualitative analysis. Taken together, the categories make the case that HIVST and PrEP use serve to empower participating SW and their intimate partners. Each category describes a different way in which HIVST and PrEP were experienced as a bridge to empowerment by study participants. The first category shows the positive impact of HIVST and PrEP on earning capacity and income for SW. The second shows how HIVST and PrEP benefitted relationships. The third explains how HIVST and PrEP use promoted sexual health. Overall, SW experienced HIVST and PrEP as strengthening their business and intimate relationships while safeguarding sexual health.

#### 3.2.1 Category 1: HIVST and PrEP form a bridge to economic empowerment

Condomless sex is more expensive than sex with a condom. SW desired the larger sums of money offered by clients who were willing and able to pay for condomless sex, but they were fearful of acquiring HIV. HIVST and PrEP use offered a bridge across this impasse, by eliminating unknown client HIV status as a barrier to sex without condoms and providing HIV protection in the context of condomless sex. This empowered SW to generate four to five‐fold higher earnings in what an FSW called a ‘sure deal of having unprotected sex after testing’.

I do not have a problem giving unprotected sex to someone I have tested and is negative. A regular customer called me and [offered] 150,000 shillings [$39.65] for unprotected sex. When he came, I tested him, and he was negative. I knew that I have taken my medicine [PrEP], I will make this money. We had unprotected sex as he wanted, and he gave me my money. (FSW, age 27)

‘I used to have protected sex with him, but he wanted live [condomless] sex. I asked him, ‘Do you accept to test for HIV before we can have live sex?’ He said, ‘Yes’. I confirmed that he is HIV‐negative. After that he told me, ‘Now we are going to start having live sex’. I [asked for] 100,000 shillings [$26.43] a night. He told me, ‘No I will be giving you 80,000 shillings [$21.15]’. After we had live sex for three different days, he told me that I gave him courage to test for HIV. (FSW, age 28)

Limited availability of HIVST kits decreased the potential for income generation and led to client stratification, a two‐tiered system in which SW prioritized test kits for better paying clients. They used the novelty of HIVST as an entry point for introducing HIV testing. Knowledge of their own HIV status and knowing how to perform and interpret self‐test results gave SW bargaining power over clients without this knowledge. There was no incentive to use limited supplies of test kits for testing clients offering lower sums of money. The absence of a relationship and lack of time to discuss testing, coupled with low earnings, made it easier to just insist on condom use for these clients.

If it is a one‐day client, that one I do not test him, I just put a condom on him, he does what he wants and goes. I do not get many self‐testing kits so I cannot test them every time they come. I test regular customers because I have time to talk to them and they also want to test. We cannot have unprotected sex if we have not tested. I find it easy to test them because they want to have sex with me, and the condition is for us to first test for HIV. Once they love you, they do what you tell them to do. (FSW, age 30).

You cannot test men you have seen for the first time. You need a lot of time with somebody that you cannot have with someone new. Self‐testing kits can easily be used to test a customer that a sex worker has known for some time. It was easy for me to test such people because I could have enough time with them, and I could hold conversations with them unlike the casual clients who want sex and nothing else. Another customer that can be easily tested is the one that a sex worker is going to spend a night with. If he has money and asks for unprotected sex, I can freely give him because I know he is negative. (FSW, age 22)

Taking PrEP was empowering because it gave SW greater control over their HIV risk. This made it easier to exchange condomless sex for higher earnings. SW were willing to take a calculated risk to generate more revenue because they believed in PrEP protection. Taking PrEP meant clients offering ‘good money’ could have condomless sex even if the limited supply of test kits meant they had not been tested. Disclosure of PrEP use to intimate partners motivated them to consider taking PrEP.

Ever since I started taking PrEP I can have live sex with a man for as long as [he] is giving me good money. When I have PrEP, the only fear I have is STDs which the testing kit does not test for. I no longer worry about HIV because I have PrEP. (FSW, age 28)

My partner told me about [PrEP]. She told me that there are tablets that people take every day. Condoms breaking during sexual intercourse is the major challenge that puts my life at risk. PrEP will be of help. (cisgender man, age 48, partner of FSW)

#### 3.2.2 Category 2: HIVST and PrEP form a bridge to relational empowerment

HIVST acted as a bridge to relational empowerment by rebuilding bonds with intimate partners after periods of separation. FSW reported it was common for them to be separated from their partners for long periods when men worked away from home. Men were assumed to have had other sexual liaisons while away, leading to increased fears of HIV infection that interfered with intimacy when the couples reunite. SW tested themselves together with intimate partners to normalize HIVST; this appeared to reduce hesitancy to test by the partners. By self‐testing returning intimate partners, they were able to dispel doubts about their partner's status, overcome fear of HIV infection and renew trust in the partnership. Discovering partners were HIV‐negative was a relief to FSW, who until that moment may not have known their partner's sero‐status. The shared experience of partner testing brought couples closer together, strengthening their relationship and enabling them to enjoy intimacy without fear and suspicion. Self‐testing was empowering for partnered relationships because it created a bridge across the emotional divide resulting from separation.

The man that I have at home who spends three months away, do you think he will live without sex where he is at? You do not know whom he is sleeping with and that is why I tell you that I was so happy for this medicine [PrEP]. I wanted us to test ourselves. He tested, and the Lord helped him, and he was fine [HIV‐negative]. He was happy because he told me, ‘Protect yourself because I am protecting myself when I am out there’. (FSW, age 38)

I got a boyfriend for love purposes. I told him that we were going to test, ‘but I am going to test you myself’. He did not refuse, and he was HIV‐negative. I was happy, and we even had live sex there and then because I also wanted. It was the first time we were having live sex; we were using condoms before. (FSW, age 30)

Mutual disclosure of HIV status also helped to build trust. For returning partners also, a spouse's HIV‐negative results were viewed as a sign of fidelity. HIV‐negative test results also motivated both partners to protect themselves from HIV and stay HIV‐negative to strengthen and preserve the relationship. Testing HIV‐negative together enabled partners in an established relationship to experience sexual intimacy by shifting from sex with a condom to condomless sex.

We tested and [learnt] we are of the same status and our love for each other increased. After we used the kit, we became happier as we were both HIV‐negative. Because we trust each [other], I know that she cannot cheat on me, and neither can I cheat on her. If she tested HIV‐negative by using the HIV self‐testing kit and it is the same kit that has tested me, I am confident about my results. (cisgender man, age 27, partner of FSW)

Before testing, we always used condoms. After knowing that we are both HIV‐negative, we stopped using them. We even had more trust in each other. You lose fear and stop doubting your partner's status, because we don't think of testing immediately after getting a partner. [Testing] comes afterwards when you have spent time having sex with each other. Basically, I was strong that we are both safe; let's move on with life. (cisgender man, age 25, partner of FSW)

Taking PrEP was also experienced as a bridge to intimacy. Condoms provided a physical barrier against HIV infection but presented an emotional barrier to intimacy. With PrEP, SW had a chemical barrier against HIV, which reduced anxiety and permitted pleasure. PrEP was characterized as an ‘inner policeman’, and compared to having a security guard at home. By contrast, not taking PrEP was perceived as opening the door for HIV. Repeat negative HIV tests, despite ongoing condomless sex, reinforced the belief that PrEP worked. PrEP use was empowering because it took away fear of HIV, gave SW control over their vulnerability to HIV infection and allowed them to feel safe, protected and intimate.

You have to have your own inner policeman who fights some of your battles. PrEP has to be in my body to protect me because when I stop taking it, it can weaken…even if you have a gun inside your home, you still must guard your home because maybe the gun is [not] always there with you. When you don't take PrEP, how sure are you that you are going to be safe? (FSW, age 26)

I did not have a boyfriend before I got PrEP. When I started taking PrEP, I got a boyfriend. When I started taking PrEP, and I realized that it protects against HIV, I decided to get a man for love not as a client. (FSW, age 27)

#### 3.2.3 Category 3: HIVST and PrEP as a bridge to sexual health empowerment

Access to PrEP and having the option to self‐test partners at their convenience outside health facilities empowered SW by eliminating barriers to sexual healthcare. Seeking sexual healthcare at clinics can be awkward and inconvenient for SW, who face stigma and discrimination during clinic visits from both health workers and fellow patients. Testing at home, in private, using HIVST helped avoid stigmatizing encounters at clinics where PrEP is delivered alongside ART. Reducing the need for clinic visits became a key motivator for HIVST and PrEP use.

Obtaining HIV‐negative test results reinforced risk reduction behaviours among SW, such as enforcing and monitoring condom use throughout a sexual encounter. Self‐care interventions for sexual health (HIVST, condoms and PrEP) encouraged SW to remain HIV‐negative.

So, with the self‐testing kit the struggle of having to make a line while at hospital, being gossiped by other patients, finding the health worker with so many patients to work on is also done away with. I really love the self‐testing kit because there is no [queue at] the laboratory where people will start assuming that you have HIV/AIDS. You will do the testing at home with no disturbances. So, tell me, where will stigma come from? (TGW, age 23)

I started testing myself and now I am firm. I don't allow a customer to put on a condom. It is me who puts it on him. That started after I tested myself. Because the problem comes when he takes it off. But I hold onto it because you feel it. I became responsible and said, ‘I need to stay HIV‐negative and look after my children’. (FSW, age 27)

Sex work stigma, however, persisted despite the perceived reduction in stigma associated with seeking clinic‐based care. Most SW in this sample reported not having disclosed sex work to their intimate partners, opting for secrecy to avoid the anticipated negative consequences of disclosure. When intimate partners inadvertently discovered HIVST kits or PrEP drugs hidden by SW spouses, they were not told the reasons for taking PrEP, which aroused suspicions of deceitfulness.

I have been in a relationship with my boyfriend for four years. When he saw the drugs, he wondered why I had them. I had to explain to him everything and test him as well to know our status. I had to show him how it [HIVST] works by testing him. He was not interested in the PrEP drugs, but he started thinking that I was taking PrEP because I thought he was cheating on me. Of course, I didn't tell him that I do sex work because that is my business. (TGW, age 23)

I was scared about what my boyfriend will think if he sees me picking tablets from [the PrEP] container. I hid it, but after some time he saw the tablets. I explained that my sister works with health workers, so she keeps that medicine with me. When he asked my sister, she told him that she gives out that medicine to some people, [and] I help her to keep it. There is no way I can explain to him what PrEP does when it looks like ARVs. I know that if I tell him that PrEP is mine, he will think that I am infected. (FSW, age 27)

### 3.3 Potential gender differences in disclosure

The data hint at the possibility of gender differences in disclosure of HIV‐positive status to clients following testing with HIVST kits. In this small qualitative sample, MSM seemed to find it easier than women to disclose positive HIVST results to clients and link them to HIV care. For MSM, clients who tested HIV‐positive seemed to remind them of the risks of sex work, reinforcing the need for PrEP adherence. FSW on the other hand, tended to fear violence if they disclosed positive test results to clients. Instead, they chose to deflect and blame the test kit for malfunctioning.

He was my guy; we used to have sex and I told him that we should test ourselves as I had testing kits and he accepted. I tested him and it reacted as two lines appeared, and I told him, ‘You must be positive though I am not very sure so tomorrow let's go to the hospital and confirm’. I took him, they tested him, and it was true that he was sick [HIV‐positive] and he started on the medicine. From that day I have never missed [taking PrEP] because I realized that most of [my clients] are HIV‐positive. (MSM, age 22)

There were two lines on that stick [test device] he passed through his mouth. After that I said that ‘I am now finished, he has infected me’. I lost hope and felt so scared. I did not tell him what the results were. I told him that it got spoilt and I threw it away. Let me tell you the truth, I cannot tell someone that you are infected with HIV. I had to keep quiet for my safety because men are not easy; he may say that it is me who has infected him with HIV, and he beats me up. (FSW, age 30)

## 4 DISCUSSION

This analysis of qualitative data suggests that HIVST and PrEP serve as bridge to economic, relational and sexual health empowerment for a sample of FSW, MSM and TGW SW in Kampala, Uganda. SW increased their earnings by strategically determining which clients to test with HIVST, leveraging their bargaining power over clients unfamiliar with this HIV testing strategy. Using HIVST and PrEP enabled SW to overcome physical and emotional barriers to intimacy, preserve relationships with intimate partners and overcome the stigma and inconvenience associated with HIV testing at health facilities. These economic, relational and sexual health benefits of HIVST and PrEP were highly valued and extended beyond the known biological benefits of these self‐care interventions.

Sex work is a competitive market with buyers and sellers. SW with bargaining power apply pressure on buyers by charging higher prices or controlling availability [27]. With the availability of specific technology (HIVST), SW can increase their selling power by offering a differentiated product (e.g., HIV‐negative status), and marketing themselves as health‐conscious SW to attract and retain clientele [10]. In this study, business strategies included charging higher prices for condomless sex and requiring pre‐sex HIV testing for paying partners who neither knew how to use HIVST nor had access to test kits. This bargaining power was leveraged to increase revenue, thus enhancing economic empowerment for a marginalized population dependent on sex work to earn a living ‐ a basic human right. Although qualitative research has shown that HIVST [20, 28] and PrEP [29] are empowering for users, no published studies elsewhere have evaluated how these interventions could enhance economic empowerment. This unintended positive benefit has the potential to increase HIVST uptake among SW in sub‐Saharan Africa. However, low sensitivity of oral fluid HIVST kits in acute HIV infection, and in persons taking PrEP or ART, may result in false‐negative results and a false sense of sexual safety, thus limiting their use for decision‐making about condom use with partners [11].

SW and intimate partners participating in this study experienced HIVST and PrEP as relationally empowering. The two groups stood on opposite sides of a river of doubt about the other's HIV status, and each group had a problem, fear of HIV infection, which needed a solution. HIVST and PrEP helped to build a bridge of trust between the two sides by preserving or re‐establishing intimacy in partnered relationships and fostering relational empowerment. Like other couples, SW and their partners prioritize intimacy over the protection of using condoms [30], and condoms are less likely to be used if they are perceived to interfere with intimacy [31]. HIVST and PrEP were experienced as liberating because they provided control over HIV vulnerability [32] and opportunities for mutual disclosure of HIV status, which helped build trust in the partnership [33].

Sexual health empowerment was a key motivator of HIVST and PrEP use for qualitative study participants, because they circumvented the inconvenience, stigma and discrimination of accessing services at public clinics. With HIVST, SW can test sexual partners who would not otherwise access testing services [34, 35, 36], increasing the reach, frequency and efficiency of testing among sexual networks [37]. Future studies should evaluate strategies to enhance linkage to care for tested clients and partners with positive test results.

Sex workers and their partners experienced HIVST as convenient and private, enabling them to avoid stigma, long queues and time spent at clinics. Mutual HIV‐negative status suggested fidelity, which motivated intimacy through unprotected sex. Intimate partners interpreted PrEP use by sex workers as a sign of infidelity and lack of trust.

Qualitative methods are generally not well suited to the identification of subgroup differences; in studies with small samples, these difficulties are compounded. Our data show the complex dynamics involved in testing, interpreting and sharing test results. SW risked loss of income and violence if they disclosed HIV‐positive results. We examined different approaches to handling an HIV‐positive result depending on gender and/or partnership type. Results suggested HIVST may not always be empowering for SW, especially FSW who risked being blamed for infecting a partner following an HIV‐positive result. For MSM SW, HIVST appeared to motivate PrEP adherence when there was known HIV exposure. It also appeared to be easier for MSM to link HIV‐positive partners to care. This is a preliminary observation meriting further study.

HIVST and PrEP reinforce each other. PrEP provides protection against HIV but requires ongoing testing; HIVST facilitates repeated testing of self, partners and clients and increases agency and control. These mutually reinforcing interventions have broader economic, health and social benefits that could increase prevention uptake in this population.

Our study had several strengths. It is the first, to our knowledge, to identify empowerment as a function of joint HIVST and PrEP delivery for SW in sub‐Saharan Africa. Our qualitative sample also includes TGW, a population that heretofore has been largely hidden and invisible to HIV programming and research in Africa. SW respondents were randomly sampled to avoid selection bias. The limitations of this work include the small sample and the clinical trial setting, which may not reflect ‘real world’ implementation of HIVST and PrEP where joint delivery of these interventions is not routine. Some SW and all intimate partners were purposefully sampled; non‐probability selection may have affected analytical results. Finally, as with all qualitative researches, our results are not, nor are they intended to be, generalizable. However, they may inform programmatic delivery of these user‐controlled biomedical interventions in other African settings.

## 5 CONCLUSIONS

Self‐controlled HIV prevention interventions, including HIVST and PrEP, are being scaled up for SW in sub‐Saharan Africa and elsewhere. SW use of these interventions may not only benefit prevention efforts, but also improve quality of life in other, non‐health‐related, domains. Understanding these larger benefits, such as relational, economic and sexual health empowerment, and communicating them to stakeholders promises to strengthen uptake and use of combination prevention interventions and contribute to HIV epidemic control.

## COMPETING INTERESTS

A.M. reports a grant from Gilead Sciences, Inc. outside the submitted work. J.E.H. is a consultant for Merck. J.M.B. works for Gilead Sciences, Inc. but was faculty at the University of Washington, Seattle, USA at the time of the study. PrEP medication (FTC/TDF) was donated by Gilead Sciences, Inc.

## AUTHOR CONTRIBUTIONS

A.M., J.E.H. and N.C.W. conceptualized the study. V.K., B.K., G.K.N., A.N., C.C.T. and A.M. conducted qualitative interviews. M.A.W., T.R.M. and A.M. supervised qualitative data collection and study implementation. A.M. and V.K. read interview transcripts. A.M. and N.C.W. coded the data and developed content categories from the initial concepts. A.M. wrote the first draft along with N.C.W. J.E.H., M.A.W. and J.M.B. reviewed drafts and provided substantial edits. All authors read and approved the final version.

## FUNDING

This work was supported through research grants from the United States National Institutes of Health, grants K43 TW010695 and AI027757 (A.M.) and K24 mentor award MH114732 (J.E.H.) and P30 AI027757.

## DISCLAIMER

This paper represents the opinions of the authors and does not necessarily represent the official views of the National Institutes of Health or Gilead Sciences, Inc.

## Supporting information

Appendix S1

Appendix S2
