# Supplementary material for: HIV self‐testing and oral pre‐exposure prophylaxis are empowering for sex workers and their intimate partners: a qualitative study in Uganda
Source: J Int AIDS Soc. 2021 Sep 2;24(9):e25782. doi: 10.1002/jia2.25782 (PMC8412089; doi:10.1002/jia2.25782)
Supplement: Supplementary file 1 — Appendix S1 [file JIA2-24-e25782-s001.pdf]

Qualitative Interview Guide – Sex Partners

HIV Self-Testing to Empower Prevention Choices in Sex Workers
(Empower Study)

Version 1.3
11 April 2018

Introduction: We understand that your partner told you we were interested in hearing from you. I am talking with you to understand your experiences with testing yourself for HIV and how it influences your decisions about safer sex and HIV care. This will help us help others to protect themselves from HIV or to seek care if they have HIV.

To begin, I would like to thank you for coming. Tell me a little about yourself. Are you still in a relationship with the partner from the Empower Study? How is your relationship with her?

Now I am going to ask you about your experiences with HIV self-test kits.

1. Tell me the story of when your partner gave you the self-test kit?
   Probes: How did you feel about using the test?

2. Tell me more about the experience of testing yourself? Are there things you liked or did not like?
   Probe to find out whether he understood the instructions on how to use the kit, difficulties encountered, if he would use the kit again or recommend it to others.

3. *How did you feel when you found out your HIV status?*
   Probe for positive and negative feelings.

4. Did you disclose your HIV status to your partner? Why or why not?

5. How did you disclose your HIV status to your partner?
   Probe to get as much detail as possible.

6. What happened after learning your test result?
   Did you go to the clinic to confirm the result? Why or why not?

7. How did you feel about having to go to a clinic to confirm your test result? What happened when you went to the clinic?
   Probe to get as much detail as possible.

8. *Would you recommend the self-test kit to other men?* What would you tell/advise someone who wants to test themselves for HIV?
   Probe: Get as much detail as possible.

9. Tell me about your sexual relationships. Have there been any changes recently?

   Probe: Did self-testing change how you use condoms? In what ways? Tell me more.
   Do you and your partner(s) ever have disagreements about condom use?

10. Have there been any changes in your sexual relationship since you self-tested for HIV?

Probe to get as much detail as possible.

11. What are the ways do you think you could get HIV, given your current sexual behaviors? Tell me more…
    Probe: Ask about condom use, and whether condoms are used with some but not all partners.

12. What have you done as an individual to reduce the risk of acquiring HIV? Tell me more about that.
    Probe: Anything else you have done to reduce your risk of HIV?

13. Do you know anyone who has tested for HIV using the self-test kit? What have you heard about their experiences.
    Probe: Did they like the kit? Would they use it again? G*et as much detail as possible.*

14. After testing for HIV, did you think about taking medications to prevent or treat HIV?
    Probe: *ask about linkage to care and use of ART or PrEP.*

I do not have any more questions at this time. Is there anything else you would like to say to help us understand your experience of HIV self-testing?

Thank you very much for participating in this interview. Do you have any questions about the study or this interview, before we end.

Turn off recorder. Pay close attention to any conversation that occurs after the recorder is turned off.
