# Supplementary material for: HIV self‐testing and oral pre‐exposure prophylaxis are empowering for sex workers and their intimate partners: a qualitative study in Uganda
Source: J Int AIDS Soc. 2021 Sep 2;24(9):e25782. doi: 10.1002/jia2.25782 (PMC8412089; doi:10.1002/jia2.25782)
Supplement: Supplementary file 2 — Appendix S2 [file JIA2-24-e25782-s002.pdf]

Qualitative Interview Guide – Study Participants

Empower Study

Version 1.3
11 April 2018

Introduction: We understand that you are participating in the Empower Study. I am talking with you to understand how testing yourself for HIV influences whether or not you use condoms and/or take pre-exposure prophylaxis (PrEP) pills. This will help us help others to protect themselves from HIV using PrEP medications or condoms.

1. To begin, I would like to know: why did you decide to participate in the Empower Study?

2. Tell me more about HIV testing in this study. For example, how do you feel when you are tested for HIV in the clinic?

Probe for details. What does the respondent think about receiving HIV test results?

3. When you began the Empower study, you were given HIV self-test kits to use. I would like to know more about the last time you used the kit. What happened?

3.a. What was using the self-test kit like for you? Tell me the story of how you used it?

Probes: How did you find the instructions on how to use the kit? How did you interpret the result?

3.b. What was your reaction(s) to the HIV test result?

Probes: How did you feel when you found out your HIV status?

4. Please describe what you do to protect yourself from HIV?

Probe in detail about condom use.

5. What was different after you started self-testing?

Probe: Can you say a little more about your condom use now*?*

<u>Now I am going to ask you about HIV self-testing for your sexual partner(s)</u>

6. If you have a regular partner/boyfriend/husband, tell me the story of how you introduced the self-testing kit to your primary partner. What happened?

Probes: How did you decide the time was right to give the kit to your partner?

7. After you gave the kit to your partner, what happened? How did he react when given the kit?

Probes: Tell me more about what happened?

8. Did your partner tell you their HIV self-test result?

Probes: Tell me what happened. What did he say?

9. To your knowledge, what did your partner do after testing? Tell me what happened.

Probe: After the self-test, did he/she go to the clinic for a confirmatory test?

10. Since we have talked about your regular sexual partner, tell me about the last time you gave a client a self-test.

Probes: Does testing occur before sex?

11. In what ways has self-testing your customer(s) influenced your decisions on whom to have sex with?

Probe to get as much detail as possible. How did self-testing change your decision making around using condoms?

12. What do you hear about others sharing the test kits with other sex workers?

13. Have you ever given away a test kit?
Probe: How did that happen?

14. What happens when a customer refuses to test?

15. What happens when a customer tests HIV positive?

16. What are the things you and your customer(s) have done to reduce your risk of getting HIV through sex?
Probe to get as much detail as possible.

17. How does your sexual behavior differ with different sexual partners?
Probe: How so? In what ways?

<u>Now I am going to ask you about HIV self-testing and taking PrEP pills.</u>

18. Tell me about how you have been swallowing the PrEP pills. Please describe how you have been testing yourself and swallowing the pills.
Probes: Why do you say you have been swallowing the PrEP pills that way? Tell me the story of self-testing occur before opening a new PrEP bottle?

19. How well do you think you have been taking the pills?
Probes: Ask about reminders and activities of daily living that could remind to take PrEP.

20. What challenges do you face in taking the pills? Tell me about some of them.
Probe to get as much detail as possible.

21. When was the last time you did not take the pills as you were supposed to? What happened?
Probes: What were the circumstances around the missed dose(s). What did you after finding out you had not taken the dose?

22. In what ways does testing yourself affect the way you take your pills?
Probes: In what ways to your HIV results make you want to keep taking the pills?

23. What would you tell/advise someone finding it difficult to taking their pills?
Probe to get as much detail as possible.

24. In this study, you were given a special pill container. What has been your experience in using the special pill container?
Probe to get as much detail as possible about experiences with the Wisepill device.

26. Is there anything else you would like to say to help us understand your experience of self-testing and/or taking study pills?

I do not have any more questions at this time. Thank you very much for participating in this interview. Do you have any questions about the study, or the interview, before we end?

Turn off recorder. Pay close attention to any conversation that occurs after the recorder is turned off.
